# Proteomic De-Regulation in Cyanobacteria in Response to Abiotic Stresses

**DOI:** 10.3389/fmicb.2019.01315

**Published:** 2019-06-12

**Authors:** Piyoosh Kumar Babele, Jay Kumar, Venkatesh Chaturvedi

**Affiliations:** ^1^Department of Biological Sciences, Indian Institute of Science Education and Research Bhopal, Bhopal, India; ^2^School of Biotechnology, Institute of Science, Banaras Hindu University, Varanasi, India

**Keywords:** cyanobacteria, abiotic stress, proteomics, PTMs, up-regulated protein, down-regulated protein

## Abstract

Cyanobacteria are oxygenic photoautotrophs, exhibiting a cosmopolitan distribution in almost all possible environments and are significantly responsible for half of the global net primary productivity. They are well adapted to the diverse environments including harsh conditions by evolving a range of fascinating repertoires of unique biomolecules and secondary metabolites to support their growth and survival. These phototrophs are proved as excellent models for unraveling the mysteries of basic biochemical and physiological processes taking place in higher plants. Several known species of cyanobacteria have tremendous biotechnological applications in diverse fields such as biofuels, biopolymers, secondary metabolites and much more. Due to their potential biotechnological and commercial applications in various fields, there is an imperative need to engineer robust cyanobacteria in such a way that they can tolerate and acclimatize to ever-changing environmental conditions. Adaptations to stress are mainly governed by a precise gene regulation pathways resulting in the expression of novel protein/enzymes and metabolites. Despite the demand, till date few proteins/enzymes have been identified which play a potential role in improving tolerance against abiotic stresses. Therefore, it is utmost important to study environmental stress responses related to post-genomic investigations, including proteomic changes employing advanced proteomics, synthetic and structural biology workflows. In this respect, the study of stress proteomics offers exclusive advantages to scientists working on these aspects. Advancements on these fields could be helpful in dissecting, characterization and manipulation of physiological and metabolic systems of cyanobacteria to understand the stress induced proteomic responses. Till date, it remains ambiguous how cyanobacteria perceive changes in the ambient environment that lead to the stress-induced proteins thus metabolic deregulation. This review briefly describes the current major findings in the fields of proteome research on the cyanobacteria under various abiotic stresses. These findings may improve and advance the information on the role of different class of proteins associated with the mechanism(s) of stress mitigation in cyanobacteria under harsh environmental conditions.

## Introduction

Cyanobacteria are the first oxygen evolving organisms which have analogous photosynthesis machinery to higher plants and encompass the ability to fix atmospheric carbon dioxide (CO_2_) and produce oxygen (O_2_). The aerobic life on the planet came into existence after the evolution of cyanobacteria. They flourished during the period from 2.8 to 3.5 billion years ago, provided Earth with oxygen and made possible the development of diverse forms of lives (Castenholz, 1996; Fischer, 2008). Cyanobacteria universally inhabit in almost all ecosystems including the Arctic and Antarctic region (Stanier and Bazine, 1977). They also occur in extreme environments, such as high salinity, pH and light irradiances (Castenholz, 1996). Cyanobacteria significantly contribute to the biomass production on the Earth by playing a potential role in the important biogeochemical cycles (e.g., nitrogen, carbon, and oxygen) (Häder et al., 2007). Presence of metalloenzymes known as nitrogenases makes them an excellent natural nitrogen fixer, and therefore they are utilized as biofertilizers in paddy and other nitrogen deficient crop fields (Vaishampayan et al., 2001; Chatterjee et al., 2017). They are massively exploited in biotechnological and pharmaceutical fields because of a unique repository of useful bioactive molecules (Rastogi and Sinha, 2009). They are an excellent source of renewable energy and bio-hydrogen, also genetically engineered for the production of bio-hydrogen and bio-ethanol which can be used as a substitute for conventional sources of energy, as it is clean, safe and environmental friendly (Khetkorn et al., 2017). Many other species are the rich resource of vitamins and minerals therefore used as food and feed supplements (MišurCoVá et al., 2010).

It is believed that the plastids of today’s photosynthetic eukaryotes including plants are the product of an endosymbiotic event between a eukaryotic host and a cyanobacterium (de Vries and Archibald, 2017). This hypothesis proved cyanobacteria as a useful model to study the stress responses, which can be directly or indirectly correlated with higher eukaryotic photosynthetic cells including plant systems (Burnap, 2015). However, there are plant processes such as organ level organization, where cyanobacterial models are not useful. Several molecular approaches are used to investigate the physiology and metabolism of a stressed cell at the DNA, RNA, and protein levels but it is not validated that how transcript and protein expressions link with the specific cellular networks (Gao et al., 2009). Furthermore, it was reported that experimental outcomes of expression levels of mRNA and proteins are contrasting (Zhang et al., 2009). It has been elucidated that the de-regulation in gene expression is not a direct approach to elucidate and understand the precise molecular machinery associated with the different stress-induced responses (Hongsthong et al., 2009; Kurdrid et al., 2011). Proteomics offers advancement to understand the cellular functions because it can crosslink the actual functions of genes, and its translational products and can reflect the cellular protein profile under defined stress conditions (Li et al., 2011). Proteomics can explore the structure and function of proteins by functioning as a connecting link between the transcriptomic and metabolomic profile, thus dissecting the actual physiological and metabolic state of the cells. Furthermore, in a stressful condition, long-term cellular adaptation is governed by the synthesis of “adaptation proteins.” These adaptation proteins are investigated to delineate the mechanism of cellular adaptation under the stress conditions. Identification of deregulated proteins using high throughput proteomics tools (gel based and gel free labeled based approaches) has provided new insights in the field of stress proteomics research. Identification and characterization of deregulated proteins provide necessary information regarding the cellular response of cyanobacteria to a defined stress condition at the level of their functions and therefore advance our understanding toward the cell signaling and stress response pathways that are activated under stress conditions. Several studies on proteomics have been successfully conducted on different cyanobacterial species under various abiotic stresses (Table 1). Literature survey suggests that a huge array of reviews on the physiology of cyanobacteria under stress conditions are available (Castielli et al., 2009; Latifi et al., 2009) but articles specifically dealing with the protein deregulation under abiotic stresses are limited in number. In this review, we made an attempt to describe several stress-responsive proteins differentially expressed in different cyanobacteria under various stress conditions.

**Table 1:** A summary of articles published dealing with cyanobacterial proteome analyses in response to various abiotic stresses.

Cyanobacteria	Stress	Methodologies	Proteins identified	Major findings	References
*Synechocystis* sp. PCC 6803	Acid stress	2-DE coupled with MALDI-TOF MS and LC-MS/MS	45	14 novel proteins with unknown functions were reported in periplasm having significant changes in response to pH. Study provides ideal targets for further studies in understanding pH stress response in cyanobacteria.	Kurian et al., 2006
*Synechocystis* sp. strain PCC6803	Salt stress	2-DE and MALDI-TOF MS. Immuno-blot-analysis.	109	Many periplasmic proteins were enhanced/induced and characterized as binding proteins of ABC-transporters or hypothetical proteins. FutA1 (Slr1295) and Vipp1 (Sll0617) exhibited the highest enhancement during stress. These are found to involve in protection of photosystem II under iron deficiency and in thylakoid membrane formation, respectively. Other proteins are regulatory proteins such as PII protein, LrtA, and a protein that belongs to CheY subfamily.	Huang et al., 2006
*Synechocystis* sp. strain PCC6803	Salt Stress	2-DE and MALDI-TOF MS and RT-PCR	337	Fifty-five proteins were up-regulated/accumulated by salt shock or after long-term salt acclimation. Some of the proteins are salt stress-specific, while other proteins are associated in general stress acclimation. In particular, heat-shock and ROS scavenging proteins are over expressed. Enzymes involved in basic carbohydrate metabolism were deregulated. Transcriptome analyses revealing that 89% of the proteins induced shortly after salt shock were also found to be induced at the mRNA level.	Fulda et al., 2006
*Synechocystis* sp. PCC 6803	UV-B stress	2-DE and MALDI-TOF MS	112	Identified proteins are classified as protein of amino acid biosynthesis, photosynthesis and respiration, energy metabolism, protein biosynthesis, cell defense, and other functional groups. Cell defense and other function proteins are involved in stress mitigation. Protein associated in photosynthesis, respiration and energy metabolism are severely affected. Study reveals the correlation between UV-B stress-responsive proteins and the physiological changes.	Gao et al., 2009
*Euhalothece* sp. BAA001 and *Synechocystis* sp. PCC 6803	Salt stress	SDS-PAGE and iTRAQ	243	Extremely halotolerant and moderately halotolerant cell were compared for the relative protein abundance for similar salt concentrations. Proteomic analysis have revealed that both the cells shared similar strategies for their survival strategies by the up-regulating higher number of “stress” related proteins in response to the salt stress. *In vivo* metabolic labeling experiments, by iTRAQ successfully demonstrated its applicability in cross-species proteomics.	Pandhal et al., 2008a
*Synechocystis* sp. PCC 6803	High pH stress	2D and 1D gels and MALDI-TOF MS	55	Comparative proteomic analysis reveals that 25 proteins were enhanced/induced and 14 were reduced in high pH condition. Most of these proteins are classified as transport and binding proteins of ABC transporters including 3 phosphate transport proteins. Other proteins include MinD involved in cell division, Cya2 in signaling and proteins involved in photosynthesis and respiration. Among these identified proteins, eight were found to be hypothetical.	Zhang et al., 2009
*Spirulina platensis*	Low temperature	2D-DIGE, MALDI- TOF MS.	Plasma membrane-40; Soluble- 2; and Thylakoid membrane-30	Differential protein expression analysis revealed that proteins related to two-component response systems, DNA repair, molecular chaperones, stress-induced proteins and proteins involved in other biological processes such as secretion systems and nitrogen assimilation were significantly up-regulated proteins in each sub-cellular fraction. In the thylakoid membrane fraction the chlorophyll biosynthetic proteins (protochlorophyllide oxidoreductase and ChlI), had unique expression patterns their expression levels significantly increased after low-temperature exposure, indicating the importance of the chlorophyll biosynthesis in response to low-temperature stress under the light condition.	Hongsthong et al., 2008
*Spirulina platensis*	High temperature	2D-DIGE, MALDI- TOF MS. Western blot, and RT-PCR	Soluble-8; Plasma membrane-52 (PM) and Thylakoid membrane-39	Proteins involved in two component response systems, DNA damage and repair systems, molecular chaperones, known stress-related proteins, and proteins involved in other biological processes, such as capsule formation and unsaturated fatty acid biosynthesis were significantly up-regulated in PM fraction. The clustering of all differentially expressed proteins in the three cellular compartments showed the majority of the proteins in all fractions were sustained tolerance proteins, suggesting the roles of these proteins in the tolerance to high temperature stress.	Hongsthong et al., 2009
*Spirulina platensis*	Low and High Temperature	iTRAQ, LC-MS, RT-PCR, Y2H system		Low-temperature stress is tightly linked with oxidative stress as well as photosynthesis. While High temperature stress was revealed to be linked with nitrogen and ammonia assimilation. Results indicate the role of cross-talk of cell signaling pathways. Hik14, Hik26, and Hik28, are the important signaling proteins, they have potential interactions with other deregulated proteins identified in both temperature stress conditions. The Y2H results obtained in this study suggests that the potential PPI network gives quite reliable potential interactions for *Spirulina*.	Kurdrid et al., 2011
*Synechocystis* hik33-knockout mutant	Salt	2D-DIGE MALDI- TOF	26	Major changes, due to the Hik33 mutation, included the substrate-binding proteins of ABC transporters, such as GgtB and FutA1, regulatory proteins including MorR and Rre13, as well as several hypothetical proteins. Under salt stress conditions, the Hik33 mutation reduced levels of 7 additional proteins, such as NrtA, nitrate/sulfonate/bicarbonate-binding protein, LexA, and enhanced levels of 9 additional proteins including SphX. These observations suggest a substantial rearrangement in the plasma membrane proteome of *Synechocystis* due to the loss of hik33.	Li et al., 2011
*Anabaena* sp. PCC 7120	Arsenic	2-DE, MALDI-TOF/ MS, RT-PCR	45	Protein involved in metabolic and antioxidative defense were significantly up-regulated. Phytochelatin and GST work synchronously and the *ars* genes play a central role in detoxification and survival of cells under arsenic stress.	Pandey et al., 2012
*Synechocystis* sp. PCC 6803	Hexane	iTRAQ LC-MS/MS	1,492	Functional annotation and KEGG pathway enrichment analyses showed that common stress responses were induced by hexane. Notably, a large number of transporters and membrane-bound proteins, proteins against oxidative stress and proteins related to sulfur relay system and photosynthesis were induced, suggesting their possible major role in the protection mechanisms against hexane toxicity.	Liu et al., 2012
*Arthrospira (Spirulina) plantensis-YZ*	Salt	2DE, MALDI-TOF/ TOF, qRT-PCR	141	Study describes de-regulation in 114 proteins showing homology with *Arthrospira* and other bacterial species. Most of the identified proteins showed consistency at both transcription and protein levels. Differentially expressed proteins were classified into 18 types of functional categories using COG database, and linked them to their respective KEGG metabolism pathways. These proteins are involved in 31 metabolic pathways, such as photosynthesis, glucose metabolism, cysteine and methionine metabolism, lysine synthesis, fatty acid metabolism, glutathione metabolism. Additionally, the SRPs, heat shock protein and ABC transporter proteins were identified, which probably render resistance against high salt stress.	Wang et al., 2013
*Synechocystis* sp. PCC 6803	Butanol	iTRAQ and LC-MS/MS	303	Reported that butanol exposure to the cells led a significant decrease in overall primary metabolism, modification of cell membrane and envelope, and initiation of oxidative stress response causing an increase in specific protein induction for fighting stress. Significant changes in the induction of heat-shock, transport and membrane/envelope modification proteins suggested that they could be the major defense response proteins during butanol stress. These proteins could be the potential target genes/proteins for further metabolic engineering to generate stress-resistant hosts for fuel production.	Qiao et al., 2012; Tian et al., 2013
*Synechocystis* sp. PCC 6803	Cadmium, nickel and cobalt	2-DE MALDI TOF/MS RT-PCR	20 (Nickel) 26 (Cobalt) 13 (Cadmium)	Results have shown that some proteins are commonly regulated in response to the different metal ions, including ribulose1,5-bisphosphate carboxylase and the periplasmic iron-binding protein FutA2, while others, such as chaperones, were specifically induced by each metal. Carbon metabolism and photosynthesis are the main processes severely affected by the metals since heavy metals affect proteins required for the correct functioning of these activities.	Mehta et al., 2014
*Anabaena* PCC 7120, *Anabaena* L-31 and *Anabaena doliolum*	Methyl viologen	2DE, MALDI-TOF MS/MS	103, 92, and 41, respectively	The proteomic response, at respective LD_50_ dose, was investigated and oxidative stress-responsive proteins were identified and compared with methyl viologen responsive proteins reported from *Anabaena* 7120.	Panda et al., 2014; Panda et al., 2015
*Anabaena* L31, *Anabaena* sp. PCC 7120 and *Anabaena doliolum*	UV-B stress	2DE, MALDI-TOF MS/MS	90, 91 and 98, respectively	In *Anabaena* L31 operation of an alternate pathway for assimilation of nitrogen and carbon under UV-B stress. An early accumulation of four proteins viz., glutamate ammonia ligase, transketolase, inorganic pyrophosphatase and trigger protein could be used as a stress biomarker of UV-B stress in *Anabaena* species.	Shrivastava et al., 2015
*Anabaena* sp. strain 90	Inorganic phosphorus (Pi)	2D-DIGE and LC-MS/MS	43	Correlation analysis unraveled an association only to some extent between the transcriptomics and proteomics abundances. Results suggest that the method used for monitoring the Pi status in cyanobacterial bloom should contain wider combinations of phosphate regulation genes (e.g., PstABCS transport systems) in addition to the commonly used alkaline phosphatase gene alone.	Teikari et al., 2015
*Arthrospira* sp. PCC 8005	Gamma rays	2DE, MALDI-TOF, Microarray		Results showed a decline in photosystem II quantum yield, reduced carbon fixation, and reduced pigment, lipid, and secondary metabolite synthesis. In contrast, there was induction in the transcription levels of photo-sensing and signaling pathways, and thiol-based antioxidant systems genes and proteins.	Badri et al., 2015
*Anabaena* sp. strain L31	UV-B	2DE, MALDI TOF MS/MS, Bioinformatics	21	Twenty one proteins, including two hypothetical proteins (HPs) were identified and placed in eight functional categories. However, several of the proteins were housekeeping proteins involved in key metabolic processes such as carbon, amino acid biosynthesis and energy metabolism, certain proteins seem to have a role in stress (antioxidative enzymes), translation, cellular processes and reductases. Two novel HPs (all3797 and all4050) were characterized in detail. These two were over-expressed after UV-B irradiation and characterized as FAS 1 (all3797) and PRC barrel-like (all4050) proteins. Bioinformatics analysis revealed that the genes of both the HPs have promoter regions as well as transcription binding sites in their upstream region (UTR). Promoters present in all3797 genes suggest their crucial role against UV-B and certain other abiotic stresses.	Babele et al., 2015
*Synechococcus* (tropical strain WH8102 and temperate strain BL107)	Low temperature	Label-free shotgun proteomics, qRT-PCR		Global proteomic profiling demonstrates the classification of the proteome into major categories: photosynthesis (44%), translation (10–15%) and membrane transport (2–8%) with distinct differences between and within strains grown at different temperatures. At low temperature, growth and photosynthesis of strain WH8102 was significantly depressed, while BL107 show negligible changes. There was an increased abundance of proteins involved in protein biosynthesis at 18°C for BL107. Each strain showed distinct differences in lipid composition with higher unsaturation in strain BL107. Study describe that strain BL107 has better survival ability under low temperature stress because it can better acclimatize by maintaining the membrane fluidity, abundance of protein biosynthesis machinery and the maintenance of photosynthesis efficiency. Additional stress responsive proteins unique to BL107 may also contribute to this strain’s improved fitness at low temperature.	Varkey et al., 2016
*Synechocystis* sp. PCC 6803	3-hydroxypro pionic acid (3-HP)	iTRAQ-LC–MS/MS LC–MS-based targeted metabolomics	2264	Protein deregulation analysis showed that 204 and 123 proteins were up-regulated and down-regulated, respectively. The proteins related to oxidative phosphorylation, photosynthesis, ribosome, central carbon metabolism, two-component systems and ABC-type transporters along with the abundance of 14 metabolites related to central metabolism were up-regulated. RT-qPCR analysis confirm the proteomic and metabolomic results by showing over-expression of selected genes related to three transporter genes putatively involved in cobalt/nickel, manganese and phosphate transporting (i.e., sll0385, sll1598, and sll0679) could lead to an increased 3-HP production. The results suggested that the supply of ATP and NADPH was increased significantly, and the precursor malonyl-CoA and acetyl-CoA may also be supplemented when 3-HP was produced at a high level.	Wang et al., 2016
*Anabaena* sp. strain PCC 7120 and *Anabaena* sp. strain L-31	Uranium	2DE, MALDI-TOF,		Both species displayed significant differences in levels of proteins associated with photosynthesis, carbon metabolism, and oxidative stress alleviation, commensurate with their uranium tolerance. Higher uranium tolerance of *Anabaena* sp. strain L-31 could be attributed to sustained photosynthesis and carbon metabolism and superior oxidative stress defense, as compared to the uranium sensitive *Anabaena* sp. strain PCC. Revealed that rapid adaptation to better oxidative stress management, and maintenance of metabolic and energy homeostasis underlies superior uranium tolerance of *Anabaena* sp. strain L-31 compared to *Anabaena* sp. strain PCC 7120.	Panda et al., 2017

## Adaptation Mechanism in Cyanobacteria Under Abiotic Stresses

Stress can be defined as an external factor, which exerts an internal damaging impact on the living organisms. It can also be defined as a significant variation of the most favorable condition of life (Latifi et al., 2009). The stress concept is described according to physiological and ecological requirements of an organism throughout its life-cycle. During the past several years, immense progress in industrialization and anthropogenic activities have resulted in an increase in different types of pollutants which changes the actual environment of the organisms (Bais et al., 2015). At present, abiotic stresses are one of the main concerns imposing a global challenge among us. Cyanobacteria are continuously exposed to various types of abiotic stresses such as solar ultraviolet radiation (UVR), variations in the external environment including light intensity, temperature (high and low), salinity, pH (acidic and basic), heavy metals, drought, and chemical fertilizers in their natural habitat. The growing numbers of cyanobacterial genome sequencing projects of newly identified species from diverse sources have significantly contributed to the modernization of research on cyanobacteria. In addition to conventional biology, advanced molecular biology, computational biology, and system biology methods/techniques have also contributed significantly in co-motivation in the development of high throughput methods to answer these biological questions globally. These technical advancements were made to address various key “-omic” aspects (e.g., genomics, transcriptomics, proteomics, metabolomics, and epigenomics) in accordance with the central dogma of the cellular biology. DNA microarrays are used for transcriptome profiling of organisms under a defined condition, it has been approved as a modern approach to study the deregulation in gene expression at system-level (Hernández-Prieto et al., 2014). Aided with, high throughput proteomic approaches have offered insightful advancements for the study of the function, integration, and regulation of proteome with the recent breakthroughs in biotechnology (Ow and Wright, 2009; Chardonnet et al., 2014). Following this, researchers are utilizing a wide range of high-throughput proteomic techniques to explore one of the most significant loop-hole, i.e., protein redox chemistry and flux analysis. Redox proteomics and fluxomics constitute crucial information about photoautotrophic gene regulation by signaling processes and thus metabolic rearrangements (Ansong et al., 2014; Guo et al., 2014; Diamond et al., 2017).

**FIGURE 1:**
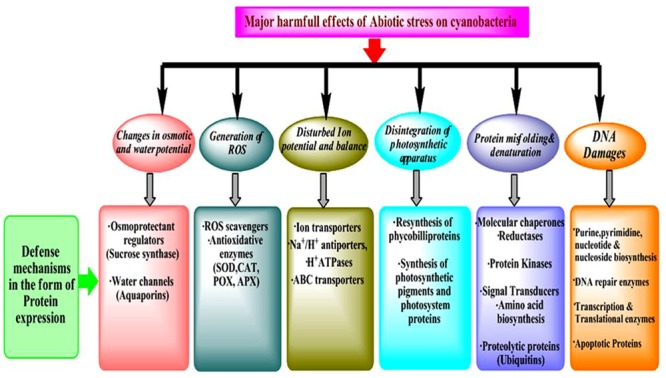
Categorization of proteins expressed at different molecular level against abiotic stress.

Accuracy and efficacy of protein biosynthesis are very crucial for life since a high degree of dependability of translation of the genetic information is essential to achieve the requirements of the cellular functions and also to preserve the variability developed by evolution. Tolerance to stress is regulated through profound de-regulation in gene expression which leads to changes in the downstream process of the central dogma. Therefore, the analysis of changes in proteome and metabolome is very important since they are direct effectors of stress responses (Burnap et al., 2015). Proteins/enzymes play essential roles inside the cell such as they catalyze various metabolic reactions, function as the components of transcription and translation machinery, and regulate stress response at metabolome levels (Hihara et al., 2001). Furthermore, proteins also have direct functions in the acclimation of stresses leading to changes in the cell physiology and metabolism (Figure 1). As discussed above, a change in protein abundance under stress is an effect of how the cells sense and respond to a particular stressor and most often determines the tolerance to stress. Therefore, investigation of post-translational modifications (PTMs) and identification of novel redox proteins as well as functional sites in the redox-regulated proteins are necessary (Foyer and Noctor, 2009). Elucidation and analysis of the redox regulation mechanisms and its effects on the role of protein along with the distinct pathways in response to environmental stresses will open new avenues in the stress biology of cyanobacteria. These studies considerably contribute to understanding the fundamental physiological mechanisms adopted by cyanobacteria to tolerate the stress responses (Paulsen and Carroll, 2013; Mo et al., 2015).

## Post-Translational Modifications (PTMs) in Cyanobacteria

To adapt and survive in a variety of stressful and ever changing conditions, cyanobacteria have evolved intricate signal transduction machinery to sense changing environmental signals. The post-translational modifications (PTMs) systems have vital regulatory role in the signal transduction pathways of cyanobacteria. PTMs are defined as the covalent modifications of proteins. These modifications occur by a number of mechanisms such as protein splicing, and phosphorylation, glutathionylation, and acetylation of specific amino acids, which lead to changes in properties and functions of a protein (Hart and Ball, 2013; Olsen and Mann, 2013; Xiong et al., 2015a). A number of proteins undergo PTMs in response to various abiotic stress signals and are reported in a number of processes in cyanobacteria, such as, photosynthesis (Xu et al., 2001), nitrogen fixation (Gallon et al., 2000), and regulation of circadian rhythms (Axmann et al., 2014). The systematic investigation of PTMs could contribute to the comprehensive description of different types of proteins and to elucidate potential biological roles of each protein in cyanobacteria. Although the proteomic studies of PTMs carried out in cyanobacteria are limited in number, but recently these investigations are gaining much attention. PTMs data have provided clues to elucidate the complex signaling mechanisms that contribute to their evolutionary and ecological success (Xiong et al., 2015a). Among these modifications, phosphorylation of target proteins is well studied mechanism and is reported in cyanobacteria grown under stress conditions (Yang et al., 2013). A TiO_2_ enrichment and LC-MS/MS based global and site-specific phosphoproteomic analysis of 245 different proteins of *Synechococcus* sp. PCC 7002 identified 410 phosphorylation sites on 280 phosphopeptides. The characterized phosphoproteins were found to be involved in numerous cellular metabolic processes such as two-component signal transduction pathway and photosynthetic reactions (Yang et al., 2013). Recently, it was studied that Ser/Thr/Tyr kinases and phosphatases also play important role in regulating a number of processes such as photosynthesis and carbon, nitrogen metabolism in cyanobacteria (Spät et al., 2015). The regulatory protein PII (GlnB) involved in nitrogen metabolism, which senses changes in the internal N/C ratio, is the most widely studied and known phosphoprotein in cyanobacteria (Forchhammer, 2008). Mikkat et al. (2014) have utilized 2D gel electrophoresis and a phosphoprotein-specific dye to report the complete protein phosphorylation profile in Synechocystis 6803 (Mikkat et al., 2014). They observed that 32 proteins were phosphoproteins undergoing PTM when cultivated under high- and low-salt concentrations. Another study on direct shotgun membrane phosphoproteome in *Synechocystis* was studied using gel-based phosphoprotein staining and in-gel digestion leading to identification of 33 phosphoproteins, including 11 membrane bound proteins undergoing PTM (Lee et al., 2015). A global phosphoproteomic profiling of Synechocystis 6803 utilizing TiO_2_ enrichment of the phosphopeptides, followed by LC-MS/MS, revealed 367 phosphorylation sites on 190 proteins. The characterized proteins were involved in different cellular functions, including photosynthesis-related proteins proposing that phosphorylation of Ser, Thr, and Tyr residues might be involved in the metabolic reactions linked to photosynthesis (Angeleri et al., 2016). These studies show that phosphorylation in cyanobacteria is involved in many metabolic processes such as central carbon, nitrogen metabolism and photosynthesis. In cyanobacteria, stress induced ROS are scavenged by cellular antioxidant defense systems, which involve the redox homeostasis of cellular thiols such as glutathione, present in its reduced form (GSH). Studies have shown that *Synechocystis* 6803 possesses only one monothiol and two dithiol glutaredoxins (Grxs), which interact with different proteins resistant to toxic metals and other stresses (Sánchez-Riego et al., 2013). Chardonnet et al. (2014) utilized biotinylate doxidized glutathione (BioGSSG) and streptavidin-affinity chromatography in combination with nano LC-MS/MS to accomplish the first detailed study of *in vitro* glutathionylation in *Synechocystis* 6803. They identified potential glutathionylation sites on 125 proteins of different metabolic pathways. These studies revealed that glutathionylation is a central mechanism in cyanobacteria to shield themselves from the frequently encountered oxidative stresses caused by high light intensities or presence of toxic metal. PTMs by lysine acetylation are another important mechanism playing an important regulatory function in signal transduction (Kim and Yang, 2011). A recent universal acetylome analysis of 513 acetylated proteins on *Synechocystis* sp. identified 776 acetylation sites (Mo et al., 2015). These lysine acetylated proteins are functionally classified and most of them involved in cellular metabolism including the subunits of phycobiliproteins (phycocyanin and allophycocyanin) (Mo et al., 2015). Functional significance of lysine malonylation in *Synechocystis* sp. PCC 6803 was studied using affinity chromatography in combination with tandem mass spectrometry. This global analysis identified 598 lysine malonylation sites on 339 different types of proteins, among them 27 proteins were involved in the reactions of photosynthesis (Ma et al., 2017). Another global proteomic profiling of *Synechococcus* sp. identified 1653 acetylation sites on 802 acetylproteins involved in a broad range of biological processes including photosynthesis (Chen et al., 2017). These findings on the global acetylation and malonylation of lysine residues on a number of proteins provide new insight into the molecular mechanisms associated with the negative regulation of photosynthetic oxygen evolution in cyanobacteria. In-depth knowledge of these PTMs help in elucidating the physiological role of protein acetylation in regulation of photosynthesis. However, system-wide screening of multiple PTMs is a highly puzzling task, in circumstances where reversible PTMs are caused by a stimulus for a short period of time. Another hindrance in PTM studies is the absence of proficient enrichment strategies for the proteins which are undergoing PTM.

## Tools and Techniques Adapted for Proteome Analysis in Cyanobacteria

**FIGURE 2:**
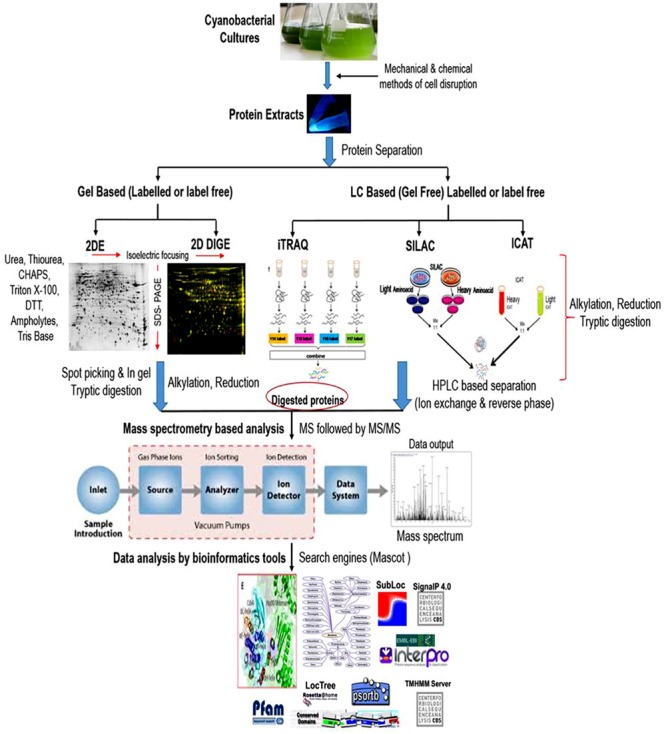
A typical workflow of comparative proteomic analysis in cyanobacteria. Proteins are extracted, cleaned and subjected to separation via gel (2-Dimensional gel electrophoresis) or non-gel (liquid chromatography) based approaches. Reduction, alkylation and trypsin digestion are performed before or after the separation step as per the requirement to convert protein mixtures into peptides. Separated peptides are analyzed through mass spectrometer (MS) followed by tandem MS (MS/MS) for the protein identification. The detected protein list is then used for data analysis using various bioinformatic tools.

Cyanobacterial proteomics till date has dealt with many technical challenges. Considerable technical advancements in the field of proteomic research have profoundly contributed to answering the complex biological questions in cyanobacteria. Gel-based proteomics has been very well adapted to analyze the proteomic alterations during growth and development, and for the investigation of proteomic responses to various abiotic and biotic insults. The most extensively used gel-based technique for protein identification is two-dimensional gel electrophoresis (2-DE), in which a large number of cellular proteins appear in the form of spots on the gel matrix and can be clearly visualized after proper protein staining. Thereafter, proteins separated on 2-D gels are preceded for digestion into smaller peptides using proteolytic enzymes and identified by mass spectrometry (MS). Although, the qualities of protein separation by gel-based techniques are debatable, but this separation strategy is extensively used with their own merits and demerits (Jorrín-Novo et al., 2015). Gel based techniques are frequently used because they are simple, reproducible, wide molecular weight coverage, and post-translational modifications detection (Mikkat et al., 2014). However, careful manual editing and optimizations of experimental conditions are required to achieve a high concentration of protein particularly for relative quantitative proteomics. Furthermore, protocols for protein extraction specific to a particular strain are also crucial considering the heterogeneity between species. Although several methods have been developed to enhance the quantity and quality of protein spots in a 2-D gel, these improvements are still not ample to describe the complete proteome profile. For example; isolation and purification of stable and high-quality proteins are one of the crucial parameters for the completion of quantitative proteomic experiments. In the case of cyanobacteria, it is the most challenging task because of their complex and robust nature (Pandhal et al., 2008a). Unlike other microorganisms, cyanobacteria possess a high amount of proteases and oxidative enzymes posing difficulty in extraction of stable proteins. An additional drawback is the abundance of phycobiliproteins and other pigments, which interfere with protein fractionation, thus hinder the effective extraction, purification, and recovery of low abundant protein posing problems in downstream analyses (Ow et al., 2011). Therefore, this technique is not appropriate for comprehensive high-throughput protein functional characterization (Qiao et al., 2012; Guo et al., 2014). Although some of these concerns have been optimized by addition of several steps in protein extraction as well as purification processes, but gel-based approaches are not capable to identify the low abundant proteins expressed exclusively in response to a particular stress (Parimi et al., 2015). Again several proteins undergo one or more post-translational modifications (PTMs), many of which are and could play critical roles in numerous cellular processes cannot be characterized by utilizing gel-based strategies due to its narrow range of pI (isoelectric point) coverage. To tackle these problems several gel-free proteomic techniques have been developed. For identification of proteins, gel-free approaches require protein digestion before chromatographic separation and mass spectrometry (MS). The number of proteins identified and quantified has primarily extended with the advancements of MS. These approaches have revealed to cover a broad range of molecular weight such as identification of proteins present in minute quantity and PTMs (Fournier et al., 2007). The better understanding on the PTMs is utmost important for the biological point of view because it can differentiate between the two identical polypeptides showing the difference in molecular mass due to a single chemical moiety [for, e.g., methyl, acetyl groups (Ghosh and Xu, 2014)]. Gel-free approaches require sophisticated instruments, but we are still facing lack of instruments that enable the assessment of each and every protein deregulated in a system as well as any PTMs at a defined set of conditions (Huang et al., 2006; Mikkat et al., 2014). Recently, gel-free isobaric tags for relative and absolute quantitation (iTRAQ) technique has grown to be more trendy and used frequently due to its better reproducibility as compared to 2-DE (Aggarwal et al., 2006). Global comparison and absolute quantification of complete protein profile due to the emergence of LC-MS based tagging approaches such as isobaric tags for relative and absolute quantitation (iTRAQ) (Stensjö et al., 2007), stable isotope labeling by amino acids in cell culture (SILAC) (Dephoure et al., 2013; Spät et al., 2015), and isotope-coded affinity tags (ICAT) (Sandh et al., 2014) have helped us to discover this field in more detail. The introduction of statistically vigorous label-free quantitative approach is also an important strategy used in quantitative proteomics research to analyze a huge number of samples (Wu et al., 2006). Therefore, the current and continuing developments in the area of mass spectrometry and structural proteomics are likely to provide better outcomes for revealing the biological processes. A typical workflow of proteomic analysis was given in Figure 2. Considerable genome sequences of many cyanobacterial strains can be accessed in the public databases but genome sequences of many important strains are still unavailable and a lot of improvements are required in this particular area. Unavailability of protein databases of unidentified species of cyanobacteria make the proteomic studies difficult but up to some extent, these hurdles are surpassed by comparing the protein sequences from the databases of model cyanobacteria (Pandhal et al., 2008b). Proteomics can successfully lead to the identification and characterization of stress-responsive proteins. Qualitative and quantitative alteration in the abundance could be associated with the physiological and metabolic rearrangements at the genotype level during stress tolerance. Different types of abiotic stresses might have changed proteomic responses. In the following section, we will describe how cyanobacteria respond against abiotic stresses mainly at the protein abundance levels and the de-regulation in proteins will be described as up- or down-regulated.

## Classification of Up-Regulated Proteins in Response to Abiotic Stress

Characterization of proteins that are up-regulated, when cyanobacteria are subjected to different abiotic stress is an essential part of the classification of stress-induced proteins and further includes the identification of the mechanisms involve against these responses. Based on previous studies on cyanobacteria, abiotic stress responsive up-regulated proteins are classified into six groups: (i) DNA repair/protection and transcription regulators (ii) Heat shock proteins (HSPs) and other stress-related proteins, (iii) cellular antioxidative enzymes (iv) proteins of lipid and other cellular metabolisms (v) Two-component system proteins and (vi) hypothetical proteins.

### DNA Repair/Protection and Transcription Regulation

DNA repair and transcription regulation mechanisms universally work for all types of living organisms and have been studied comprehensively. Several studies describe the role of many proteins linked with transcriptional regulation and DNA repair mechanisms. Studies conducted on the cyanobacterium *Anabaena* sp. revealed a higher abundance of proteins having roles in the DNA protection, and transcription like nutrient deficient-induced DNA binding proteins (Babele et al., 2015; Panda et al., 2015; Shrivastava et al., 2015). DNA-binding proteins (Dps) from starved cells have crucial functions in the bacterial defense system against oxidative stress in different cyanobacteria (Moparthi et al., 2016). By binding to DNA, Dps efficiently prevents DNA base modification and strand cleavage while not interfering with normal DNA metabolism (Martinez and Kolter, 1997). These proteins provide the defense to cells during extreme environmental conditions including nutritional deprivation. The structure and function of Dps have been a concern of several studies and reported in several cyanobacteria that possess one or more Dps proteins. Their capability to provide comprehensive defense is mainly due to three integral features of the protein; these are DNA binding, sequestration of iron, and tolerance against reactive oxygen species (ROS). Having these properties, the Dps protein family members are critical in maintaining the tolerance to oxidative stress and iron homeostasis (Shcolnick et al., 2007). During starvation, Dps play important role in gene regulation, thereby rendering the cell more resistant to cytotoxicity by regulating the expression of essential stress resistance genes (Sato et al., 2012).

DNA ligases are found to be essential for the DNA repair system. These proteins may be up-regulated during quite harsh DNA damaging conditions due to abiotic stress. When the cyanobacteria cells come under the exposure of DNA damaging agents or replication blockers (e.g., UV irradiation and high temperature), expression of regulatory proteins such as SOS initiates (Domain et al., 2004). These proteins have been known to enhance the survival of the cells by enabling augmented DNA repair capability and also by inhibiting the division of cells (Shinagawa, 1996). Transcription machinery functions as a molecular motor that traverses through genome regularly. It has been recommended that the transcription machinery, for example, DNA-dependent RNA polymerase alpha subunit (all4191) might have a potential role in detecting DNA damage which ultimately leads to the activation of DNA repair and stress-responsive mechanisms (Ljungman, 2007).

A study conducted on *Spirulina platensis* under cold stress, showed that certain proteins participating in damage, repair and modification of DNA (polymerases, exonucleases, and methylases) were many folds up-regulated in the membrane portions following temperature downshift. It is expected that the induction of SbcC will occur when DNA damage takes place. Therefore, removal of abnormal DNA forms including hairpin loops formed as a result of DNA damage will be performed by SbcC exonuclease (Darmon et al., 2007;Higo et al., 2007). In comparison with cold stress, high-temperature conditions in *Spirulina*, leads to the drastic induction of exonuclease and endonuclease, thereby explicating an elevated role in the DNA repair pathway (Dwyer et al., 2007). Chromosome segregation ATPase also showed up-regulation and contribute significantly in replication, DNA repair, and genome stability. These outcomes prove the necessity of DNA replication, modification, and repair for the survival of cells exposed to extreme cold and heat stress conditions in *Spirulina* (Hongsthong et al., 2008, 2009). Proteomic profiling of *Synechococcus* under high light stress induces the production of several enzymes and transcriptional regulators involved in the process of replication, DNA repair and modification which include ParA, GvrA, and PhrA, NusB, SigD and SYNPCC7002_A2523 as major enzyme and transcriptional regulator (Xiong et al., 2015b). Label-free comparative proteomic profiling of tropical *Synechococcus* strain WH8102 with temperate strain BL107 suggested that AbrB-like transcriptional regulators were up-regulated in both strains at low temperature condition. Three and two transcriptional regulators were found to show up-regulation in BL107 and WH8102, respectively (Varkey et al., 2016). These regulatory proteins have a significant role in acclimation during low temperature condition. A study on *Synechocystis* PCC6803 has revealed the up-regulation of AbrB, a transcriptional regulator and it might have a significant role in the uptake of carbon and nitrogen, and may also contribute to the metabolic rearrangements during low temperature stress (Kaniya et al., 2013).

### Heat Shock Proteins (HSPs) and Other Stress-Related Proteins

Molecular chaperones play significant roles in the conformational modifications (e.g., folding, misfolding, refolding, aggregation, or degradation), thus maintains the protein homeostasis and provides stress adaptability to cyanobacteria. Molecular chaperones fulfill an essential function in protein biogenesis and protein quality control in the cells. Most of the chaperones are soluble proteins, but some membrane-bound chaperones have also been recognized in cyanobacteria (Rajaram et al., 2014). Studies on *Synechococcus* sp. reported that DnaK chaperone, present on the thylakoid membrane, which help in protein translocation and translational machinery and play an prominent role in the enhancement of membrane fluidity by modulating membrane lipids under heat stress (Katano et al., 2006). Under salt stress (Fulda et al., 2006), acid stress (Kurian et al., 2006) and UV-B stress (Gao et al., 2009) in *Synechocystis* sp. molecular chaperones (GroEL, GroEs, 60 kD chaperonin 1, DnaK protein 2, and 60 kD chaperonin 1) are shown to be up-regulated. Study on *Anabaena* sp., showed up-regulation of chaperones and other stress-related proteins (GrpE, chaperonin GroEL, and DnaK type molecular chaperone) in response to UV-B stress (Shrivastava et al., 2015). Another study on *Synechococcus* sp. revealed that a number of small heat-shock proteins and many other proteases showed enhanced expression under the stress of low temperature. This observation may suggest that the protein turnover is because of either an increase in protein synthesis or induction in protein misfolding in response to low temperature (Varkey et al., 2016). The enzyme peptidyl-prolyl isomerase (a trigger factor) also known as a cold-shock protein in *Synechocystis*, acquaintances with the ribosome and assists in the proper folding of newly synthesized polypeptides by increasing the affinity of GroEL with unfolded polypeptides (Prakash et al., 2010). A study conducted on *Spirulina* sp. in response to heat shock described that glycosyltransferases were found to be up-regulated in all subcellular fractions, moreover, LysR, a membrane helicase, and ferredoxin glutamate synthase were also up-regulated in the fractions of thylakoid and plasma membrane (Hongsthong et al., 2009). Glycosyltransferase has an important role in osmo- and thermo-adaptation (Borges et al., 2004). These studies clearly demonstrate that heat shock proteins and other molecular chaperones support and care for other cellular proteins from their biogenesis, and also significantly contribute in the abolition of polypeptides that are no longer useful and may cause threat to cell viability.

### Antioxidative and Cellular Defense Reaction Proteins

Abiotic stress-induced ROS can damage cellular components and act as a stress signaling molecule (Schmitt et al., 2014). ROS generation in cyanobacteria was regulated through complex pathways such as scavenging via antioxidative enzymes for, e.g., superoxide dismutases (SOD), oxidoreductase, catalases, peroxiredoxins, thioredoxins, and AhpC/TSA (Apel and Hirt, 2004; Schmitt et al., 2014). Because of the significant accumulation of H_2_O_2_, the H_2_O_2_ detoxifying enzymes such as AhpC, catalase, peroxiredoxin showed up-regulation in the proteome map of cyanobacterium, *Anabaena* (Babele et al., 2015). The overexpression of catalases and homologs of catalase, and oxidoreductase implicit resistance to the cell against oxidative stress which is also reported in case of desiccation stress in *Anabaena* sp. (Katoh et al., 2004). As evident from the fact that AhpC scavenges intracellular H_2_O_2_ at low concentration, whereas, catalase does it at high concentration (Seaver and Imlay, 2004), however, it is also reported that high level of intracellular H_2_O_2_ inhibits AhpC resulting in the down-regulation of AhpC on different days. Under these conditions, H_2_O_2_ detoxification was governed by the up-regulation of catalase and oxidoreductase when expression of AhpC is very low. Peroxiredoxin (Prx) performs multiple functions during stress conditions; (i) can reduce H_2_O_2_ and organic hydroperoxides (Dietz et al., 2006), (ii) acting as a molecular chaperone same as HSPs (Dietz, 2011), and (iii) activators of cell signaling. It is also been reported that the level of Prx was up-regulated in maize (Requejo and Tena, 2006) and *Scytosiphon gracilis* (Contreras et al., 2010) during the arsenic and copper stress. Therefore, Prx plays an important role in the protection of cyanobacteria from abiotic stress (Hosoya-Matsuda et al., 2005). Another important antioxidative enzyme, i.e., thioredoxin (Trx) known to effectively decrease the intramolecular disulfide bridges in different target proteins (Gelhaye et al., 2005). Trx helps in the maintenance of certain cellular processes like in the reduction of ribonucleotide, suppression of cell death and also provides reducing equivalents to the antioxidant systems (Hishiya et al., 2008; Pandey et al., 2012). A study proves that it regulates hydrophobic amino acids biosynthesis in *Chlamydomonas reinhardtii* (Lemaire and Miginiac-Maslow, 2004). Thus an up-regulation in Trx may be envisioned to manage oxidative stress in cyanobacteria. These are small ubiquitous proteins having highly conserved Cys–Gly–Pro–Cys active site and are reduced by thioredoxin reductase. They have the potential to reduce the intra- or intermolecular disulfide bridges of polypeptides and associated variety of redox reactions in which electrons are transferred from NADPH to Trx reductase and finally to Trxs. These redox-based reactions strictly control many enzymatic and regulatory activities and fulfill numerous cellular functions involved in cellular responses against oxidative stress. Trxs have been reported in all cyanobacterial genomes to show expression; however, it also expresses in many bacterial species (Zeller and Klug, 2006; Pascual et al., 2010). Trxs also closely associated with other redox partners including peroxiredoxins. They tightly regulate the type II peroxiredoxins, catalase-peroxidase KatG and 1 Cys-Prx in *Synechocystis* PCC 6803 (Pérez-Pérez et al., 2009). Moreover a number of small ubiquitous proteins known as glutaredoxins (Grx-s) are also present in cyanobacteria. These enzymes maintain the cytoplasmic thiol-redox state but in contrast to thioredoxins, no oxidoreductase reported which can specifically reduce Grx-s. Instead of this, their oxidation is carried out by substrates and non-enzymatic reduction by glutathione, known as the glutathione system (Holmgren, 1989). An evolutionary study on glutathione system suggests that the phototrophic microorganisms including cyanobacteria species show presence of high levels of glutathione during stress condition (Fahey et al., 1987). The primary role of glutathione is the protection of cell during ROS-induced toxicity, this notion was proved in *Synechocystis* PCC 6803 mutants with glutaredoxin knockdown of genes, these mutants showed enhanced sensitivity against peroxides (Latifi et al., 2009). The direct cellular targets of the glutathione system and the glutaredoxins homology between different cyanobacterial species are mostly unknown and more detailed information is required.

### Proteins of Lipid Metabolisms and Transporters

Lipids are storage form of energy, they function as insulators, and are one of the most important structural components of the cell membranes with a significant role in cell signaling. They also play a vital role in stress tolerance against diverse environmental and physiological insults. Tight regulation of membrane fluidity is universal in all the living organisms for the proper functioning of biological membranes. Membrane fluidity is crucial for the mitigation and adaptation against an array of environmental stresses. Unsaturated fatty acids are an essential component in all types of biological membranes and the level of unsaturation in lipids is pertinent for controlling the membrane’s fluidity (Chi et al., 2008). Temperature (high and low) stress modulates membrane fluidity by affecting the saturation and/or unsaturation of fatty acids chains of cell membranes. Heat stress (high temperature) makes membranes more fluid by altering the hydrogen and electrostatic bonding within lipids and between polar groups of proteins, thereby transforming their architecture leading in ion leakage (Los and Murata, 1998; Morgan-Kiss et al., 2006). In contrast, cold stress (low temperature) makes membranes rigid and affect the normal functions of membrane proteins thereby inhibit solute transport of the cells by affecting the activity of H^+^/ATPase channel (Zhang et al., 2006). Polyunsaturation of membrane fatty acids is critical during the process of stress mitigation and acclimatization and help cyanobacteria to combat cold and heat stress (Singh et al., 2002). Polyunsaturation is catalyzed by the three types of enzymes known as fatty acid desaturases, among them acyl-lipid desaturases are one of important type (Gombos et al., 1992). These enzymes catalyze the incorporation of double bonds into the fatty acid hydrocarbon chains to generate the unsaturated and polyunsaturated fatty acids (Sato et al., 2004; Chi et al., 2008). They enhance the introduction of double bond in fatty acyl chains that have been esterified to a membrane glycerolipid (Sato et al., 2004). These enzymes are found on the surface of the thylakoid membranes of the cyanobacteria. The importance of desaturase enzymes and the expression of respective genes in cold stress acclimation have been well characterized for their significant roles in cyanobacteria (Wada and Murata, 1990; Gombos et al., 1992; Wada et al., 1994; Tasaka et al., 1996). It is believed that the fluidity of thylakoid membranes in cyanobacteria is controlled in such a manner that it maintain the replacement of damaged D1 protein with a newly synthesized protein by providing optimal condition (Inoue et al., 2001). Proteomic profiling of *S. platensis* culture grown under low temperature stress revealed up-regulation in (3R)-hydroxymyristoyl-[acyl-carrier-protein] -dehydratase or FabZ and an acyl carrier protein (ACP) in soluble and membranous fractions (Jeamton et al., 2008). The up-regulated FabZ protein is the first dehydratase which is a component of fatty acid elongation cycle leading to the biosyntheses of unsaturated fatty acids (Mohan et al., 1994; Heath and Rock, 1996). Furthermore, the substrate of FabZ, (3R)-hydroxymyristoyl acyl carrier protein, is located at a biosynthetic branch point which can also lead to biosynthesis of lipid. Another study on *Spirulina plantensis*-YZ documented that under salt-stress an important protein of lipid metabolism, i.e., 3-oxoacyl-[acyl-carrier protein] reductase, is up-regulated. This protein might help to promote the process of photosynthesis during salt stress by accelerating the activity of Na^+^/H^+^ transporter thus impede the expulsion of Na^+^ resulting in cell survival by preventing ion poisoning (Wang et al., 2013). Enhanced aggregation of UDP-sulfoquinovose synthase (SQD1), a protein associated with the biosynthesis of sulfolipids is also reported in two *Anabaena* species upon cadmium toxicity (Singh et al., 2015).

In the natural environment, cyanobacteria face toxicity due to heavy metals, salts, pesticides, and acids. These toxic compounds severely affect the physiology and metabolism by inducing the ion imbalance of cell. Proper ion homeostasis between intra-cellular and intra-thylakoid membrane is universal for growth and survival of a cell. To maintain the ion homeostasis transport proteins or transporters play crucial roles. These membranes bound transporters took part in the transport of ions, small or larger molecules, such as other protein, through the biological membrane. Transporters have been reported for roles against arsenic, copper, salinity, acid, heavy metal and ethanol stresses in different cyanobacteria (Zhang et al., 2015). Among these transporters, ATP binding cassette (ABC) transporters are most studied. They are associated with the transport of different types of substrates actively by using ATP hydrolysis and are located on the inner membrane (Davidson et al., 2008). Whole cell protein profile analysis of acid stress response in *Synechocystis* sp. PCC 6803 has described the role of many ion transporters. Up-regulation in the bicarbonate transporter, iron(III) dicitrate-binding protein of ABC transporter, probable extracellular solute-binding protein (slr1962), probable sodium/calcium exchanger protein (slr0681), molybdate-binding periplasmic protein were reported in the periplasmic fraction (Kurian et al., 2006). Investigation of the iron uptake protein FutA1 (Slr1295), a major iron-binding protein was found up-regulated in *Synechocystis* sp. PCC 6803 exposed to either high light or heat shock (Miranda et al., 2013). FutA1, is a part of ABC transporter concerned with the Fe^3+^ uptake by the cells. FutA1, along with its homologous FutA2 (Slr0513), play an essential role in the protection of PSII following iron depleting conditions. It was suggested that FutA1 is accountable for an effective supply of iron to the D1/D2 reaction center heterodimer and also essential for a rapid PSII repair during intense light exposure. ABC type transporters (phosphate transport) and ATP-binding protein (PstB2) were up-regulated in the three *Anabaena* species upon cadmium exposure (Singh et al., 2015). *Synechococcus* strain WH8102 displayed high expression of transport proteins in the periplasmic fraction under low-temperature stress. Increased expression of efflux transporters at low temperature could promote a defense system against accumulated toxic metabolites under stress condition. These proteins may also act as lipid transporters and modulate membrane fluidity by changing the lipid composition the at low temperature (Varkey et al., 2016). ABC transporter and cyanate ABC transporter ATP-binding-component was also significantly up-regulated in the plasma membrane and thylakoid membrane fraction of *Spirulina* sp. under low-temperature (Hongsthong et al., 2008) and in all the fractions under high temperature (Hongsthong et al., 2009) stress. Proteomic analysis of plasma membrane fraction of *Synechocystis* sp. under high pH stress describes that more than thirteen ABC type transporters were significantly up-regulated. These proteins are oligopeptide binding protein, ABC transporter subunit ycf24, ABC transporter permease protein, nitrate/nitrite-binding protein (NrtA), phosphate binding protein (PstS1), putative SbtB, phosphate transport ATP-binding protein (PstB1), and iron-binding protein (FutA1) and considered to be present in the periplasmic region of the plasma membrane. On the basis of these, it might be hypothesized that high pH stress cause nutrient deficiency, thus induce the expression of phosphate- and the ATP-binding proteins (Zhang et al., 2009). Proteomic analyses of *Anabaena* sp. strain 90 following inorganic phosphorus (Pi) stress also describe the role of several ABC transporter proteins. These proteins are significantly up-regulated upon Pi exposure (Teikari et al., 2015).

### Two-Component System Proteins

The potential of living organisms to respond against a stress condition mainly depends on its ability to sense and transduce different external and internal signals (Los et al., 2010). The structures of both sensors and regulators are modular, and numerous variations in domain architecture and composition have evolved to adapt according to specific needs in signal perception and signal transduction. In this context, the two-component systems (TCSs) are crucial as these proteins are predominantly associated with signaling pathways and they are transducers of extracellular signals. They strictly regulate the motility, production of secondary metabolites, nutrient and mineral uptake, metabolic rearrangements, and cell division etc. in a variety of bacterial species. These systems also regulate cellular physiological processes in response to environmental stresses and provide adaptation (Mitrophanov and Groisman, 2008). Two-component hybrid sensor and regulatory proteins are involved in different functions such as peptidyl-histidine phosphorylation, phosphorylation, DNA-dependent regulation of transcription, signal transduction, protein histidine kinases activity, signal transducer activity, transferase activity, transfer of phosphorus-containing groups, G-protein coupled photoreceptor activity and ATP binding activity. TCSs family proteins have three main types, these are; two-component hybrid sensor and regulators, two-component sensor histidine kinases and serine/threonine kinases. A typical TCS comprises of a sensor kinase (e.g., histidine kinase; first component) that responds to a second component known as the response regulator. Sensor kinases are usually membrane-bound proteins, having N-terminal ligand-binding domain and a C-terminal kinase domain mainly, however, certain other domains may also present. Kinases function as molecular switches which can exist in either an inactive (‘OFF’) state or an active (‘ON’) state. Upon signals recognition, they can autophosphorylate at conserved histidine residue from ATP and then activate the response regulator by translocating the phosphoryl group to a conserved aspartate residue (Stock et al., 2000). Serine/threonine kinases proteins, along with their associated phosphatases, catalyze reversible phosphorylation of protein having fundamental roles in signal transduction. Kinases generally undergo autophosphorylation and precede to transphosphorylate the downstream substrates in the presence of any external stimuli. Therefore, activity of specific target proteins was controlled by the phosphorylation of specific amino acids in two ways; directly, by inducing the alteration in active sites by modulating their conformation and by indirect regulation via protein-protein interactions (Chao et al., 2010). The activated state of kinases is strongly controlled by different mechanisms, mainly by their subcellular localizations and the binding of allosteric effectors (Huse and Kuriyan, 2002). Activation (phosphorylation) of a response regulator modulates the biochemical properties of its output domain, which can induce the downstream transcription factors and regulate the gene and protein expression.

The endurance of cyanobacteria depends on its capability to promptly respond and adapt against constantly fluctuating harsh environmental conditions. Cyanobacterial chromosomes encode 1,171 potential histidine kinases, hybrid kinases and response regulators. Like most bacteria, cyanobacteria predominantly use TCSs proteins for signal recognition, to sense and respond and for cell–cell communication signals thus regulate physiology and gene expression in response to ever-changing external environment (Grebe and Stock, 1999; Stock et al., 2000; Wolanin et al., 2002; Ashby and Houmard, 2006). Studies conducted on cyanobacterium *S. platensis* in response to low and high-temperature stress showed differential expression of TCSs protein. Certain proteins present in two-component systems for signal transduction include histidine kinase, Ser/Thr protein kinase and several response regulator, i.e., Period protein–Aryl hydrocarbon receptor nuclear translocator protein-Single-minded protein; PAC, PAS C-terminal (PAC/PAS domains) were shown to be up-regulated in *Spirulina* during cold stress. Another up-regulated protein in the plasma membrane is forkhead-associated ATP-binding cassette transporter (Hongsthong et al., 2008). This transporter protein contains a forkhead-associated (FHA) domain which can bind to the Ser/Thr kinase and therefore, necessary for phospho-dependent signaling pathways (Curry et al., 2005) and linear signaling pathways (Grundner et al., 2005). Under high-temperature stress, two-component signal transduction proteins, i.e., Ser/Thr protein kinase, histidine kinase, and response regulator domain (Gly-Gly-Asp-Glu-Phe) were also found up-regulated in the both soluble and plasma membrane fraction (Hongsthong et al., 2009). Proteome analysis of *Anabaena* L31 upon cadmium toxicity also showed up-regulation of signal transduction proteins. It includes two component response regulators such as Ycf27 and Orr-A, ABC transporter family proteins and ATP-binding protein thus signifying a signaling mechanism under cadmium stress (Singh et al., 2015). Another histidine kinase, Hik33 (NblS) was found to be involved in perceiving high temperature and many other environmental insults induced signals in cyanobacteria. This protein shows higher accumulation in a temperate *Synechococcus* strain BL107 and a response regulator (SrrA) which interacts with Hik33 was induced in tropical strain WH8102 under low-temperature stress (Varkey et al., 2016). It was documented that srrA gene is induced transiently under high light and is involved in regulating the expression of phycobiliproteins in *Synechocystis* PCC7942 (López-Redondo et al., 2010).

### Hypothetical Proteins

Apart from similarities in their amino acids composition whining and between other organisms, the functions of hypothetical proteins are generally not known. A number of hypothetical proteins showed differential abundance during abiotic stresses in different cyanobacteria. Proteome analysis of *Synechocystis* PCC 6803 showed accumulation of several hypothetical proteins such as sll1863, sll1762, sll0596, slr1485, sll1549, slr2144, slr1535, and slr0711 under salt stress (Fulda et al., 2006). Protein sll0595 has a role in signaling, slr0711 in folate biosynthesis and other protein have domains of unknown functions. Bhargava et al. (2008) have shown the role of alr0803 in Cu homeostasis in *Anabaena doliolum*. Therefore, alr0803 may act as a sensor of abiotic stress and help in maintaining the homeostasis. It was also observed that hypothetical proteins alr3199, alr0803, all1009, alr4050, all4894, were also over-expressed under desiccation stress thus create desiccation like environment inside the cells. The up-regulation of alr4050 (homolog of akinete marker) protein (Zhou and Wolk, 2002), alr3199 (homolog of HHE domain) and a cation binding protein has also been reported in UV-stressed *Nostoc commune* (Ehling-Schulz et al., 2002) and in *Nostoc* sp. strain HK-01 under dehydration like stress (Yoshimura et al., 2006). Expression of a fasciclin superfamily protein (all4894), which play important role in cell–cell adhesion and connection with the extracellular matrix, indicate that this might be involved in aggregation of cyanobacterial filaments to avoid stress exposure (Kim et al., 2000). Induction of all1009 was observed under metal stressed *Anabaena* cells. This protein has also shown similar induction under drought stress in *Anabaena* species (Yoshimura et al., 2007). Both hypothetical proteins (all4895 and all1009) are closely related with the outer membrane porin OprB (carbohydrate selective porin) of *Pseudomonas aeruginosa* and most abundantly present on the outer membrane of heterocysts (Merroun et al., 2005; Moslavac et al., 2007). It is believed that these proteins have a major role in metal sequestration and carbohydrate transport from vegetative cells to heterocyst (Moslavac et al., 2007). A sugar kinase/HSP70/actin superfamily (all5091) protein share a core structural folding this protein coupled with ATP hydrolysis causing conformational changes and thus prevent the protein misfolding by enhancing the affinity of HSP70 toward unfolded protein (Liberek et al., 1991). Another up-regulated protein is alr0893, its accumulation in *Anabaena* sp. PCC7120 under UV-B stress might be involved in nullifying the damaging effect of UV radiation with its combined ATP-independent repair activity of PfpI protease and ferritin domain (Shrivastava et al., 2015). Proteomic analysis of *Anabaena* L31 upon cadmium exposure showed up-regulation in a hypothetical protein alr0882, a homolog of universal stress protein (UspA) of *A. variabilis* ATCC 29413 and in alr0806, a homolog of high light inducible protein (HLIP) from *Synechococcus* sp. indicate roles of these in photoprotection under cadmium stress. Another up-regulated protein, i.e., alr3199, a homolog of hemerythrin/HHE cation-binding region of *A. variabilis*, exhibiting DNase activity and might have some role in DNA damage repair mechanism upon cadmium induced genotoxicity in *Anabaena* species (Singh et al., 2015). Proteomic analysis of *Anabaena* PCC7120 under salt and UV-B stress describe the up-regulation in alr0882, all5218, all3014, alr3904, and alr3090. Hypothetical proteins alr3904 and all3014 are predicted as glyoxalase II while alr3090, as catalase like, and all5218 as DNA gyrase activities. Hypothetical proteins alr3199 and alr4050 exhibited over accumulation in response to high salt stress in *Anabaena* PCC7120 (Rai et al., 2013).

## Classification of Down-Regulated Protein in Response to Abiotic Stress

Based on several studies on abiotic stresses on cyanobacteria, down-regulated proteins are mainly classified into three families (i) Enzymes of amino acid, fatty acid and cofactor biosynthesis (ii) Proteins of energy metabolism (photosynthesis, respiration, and carbon/nitrogen fixation) and (iii) Enzymes of protein biosynthesis, and transport.

### Enzymes of Amino Acid, Fatty Acid, and Cofactor Biosynthesis

Photoautotrophic growth of cyanobacteria requires a highly coordinated and regulated allocation of cellular macromolecules to diverse metabolic processes. These metabolic processes include the *de novo* synthesis of proteins, lipids, and other cellular macromolecules including cofactors. For unicellular organisms, the optimal allocation of limited resources is a key determinant of evolutionary fitness. Amino acids and fatty acids are the most essential components in all living organisms because they are building blocks of proteins and lipids. Enzymes primarily involved in amino acids, lipid and cofactor biosynthetic pathways are significantly down-regulated upon a variety of abiotic stresses. It was reported that the enzymes cysteine synthase (all2521) and D-3-phosphoglycerate dehydrogenase (alr1890) are drastically down-regulated under UV-B stress, which may negatively influence the amino acid biosynthesis pathway. In our study, we found down-regulation of D-3-phosphoglycerate dehydrogenase in *Anabaena* L31 under UV-B stress (Babele et al., 2015). Enzymatic activity of D-3-phosphoglycerate dehydrogenase catalyze the conversion of 3-phospho-D-glycerate into 3-phosphohydroxypyruvate which is the committed step in the phosphorylated L-serine biosynthesis pathway. It is also crucial for the biosynthesis of cysteine and glycine amino acids (Grant, 2012). Cysteine is one of the precursor amino acids of glutathione (GSH) biosynthesis, it is a reducing tripeptide thiol, required for maintaining balance in intracellular redox potential and also support in xenobiotic detoxification (Pickering et al., 2000). Proteins involved in amino acid metabolism (3-phosphoshikimate 1-carboxyvinyl-transferase) cofactor biosynthesis (thiamine biosynthesis protein; thiC), fatty acid and phospholipid metabolism (CTP synthetase), and cell envelope proteins (dTDP-glucose 4-6-dehydratase, glucose-1-phosphate thymidylyltransferase) are down-regulated in *Synechocystis* sp. under sustained UV-B irradiation (Gao et al., 2009). A study conducted on *Spirulina platensis* under cold shock revealed that macrolide-type polyketide synthase (fragment) and curamycin polyketide synthase acyl carrier protein (ACP) involved in lipid metabolism are many folds down-regulated. Another enzyme of lipid metabolism, i.e., Δ^9^-desaturase was down-regulated during high-temperature stress in the thylakoid and plasma membrane fraction of *Spirulina* (Hongsthong et al., 2009). Label-free shotgun proteomic analysis of marine *Synechococcuss* sp. strain WH8102 showed the down-regulation in the protein involved in coenzyme metabolism (Varkey et al., 2016).

### Proteins of Energy Metabolism (Photosynthesis, Respiration, and Carbon/Nitrogen Fixation)

Cyanobacteria are the only photosynthetic prokaryotes, have the capability to convert inorganic carbon into useful carbohydrates at the expense of solar energy. This ability provides them the potentiality to produce bio-energy and other useful biochemical products (Gudmundsson and Nogales, 2015). Photosynthesis is a multistep process of successive redox reactions that comprises a series or proteins/enzymes and accessory pigments. In cyanobacteria, photosynthetic apparatus present in thylakoid membrane usually comprises a chain of membrane-bound multi-subunit protein complexes, consisting of photosystem I (PSI), photosystem II (PSII), cytochrome (Cyt) b6f, and ATP synthase (ATPase) complexes and many small (accessory pigments; plastocyanins, plastoquinones etc.) electron transport molecules (Casella et al., 2017). The thylakoid membranes also harbor the protein machinery of respiratory electron transport chains (ETC) (Mullineaux, 2014). Several studies were performed to describe the harmful effects of abiotic stresses on the photosynthesis and central carbon metabolism (CCM). Different types of oxidative stresses promote over accumulation of ROS thus lead to irreversible damage to photosystems (PS1, PSII) (Latifi et al., 2009). In cyanobacteria, phycobiliproteins are the most abundant accessory photosynthetic pigments of PSII (approximately 40% of total proteins). They are constituted of non-covalently associated subunits of allophycocyanin, phycocyanin, and phycoerythrin and play an important role in light harvesting and photolysis of water (Bryant and Canniffe, 2018). In our study, we found that UV-B stress can severely damage allophycocyanin beta subunit, phycocyanin A subunit, phycoerythrocyanin alpha chain and down-regulate the activity of F_0_F_1_ ATP synthase (beta subunit) in *Anabaena* L31 cells (Babele et al., 2015). A comparative study on three *Anabaena* species in response to methyl viologen (Panda et al., 2015), UV-B radiation exposure (Shrivastava et al., 2015) and cadmium toxicity (Singh et al., 2015) show reduced levels of most common PSII (ApcA, CpcA, CpcG4, CpcB, and PecC) proteins and ATP synthase alpha subunit. There was many folds down-regulation in phycocyanin α chain, phycoerythrocyanin α chain and phycobilisome rod core linker proteins were observed. Label-free shotgun proteomic analysis of marine *Synechococcuss* sp. strain WH8102 describes that low temperature has a significant impact on the abundance of photosystems and phycobiliproteins (Varkey et al., 2016). D1 protein (a core subunit of PSII), cytochrome c-550 (PsbV), manganese stabilizing protein of PSII (PsbO), PSI assembly proteins (Ycf3) and ROD linker polypeptides were down-regulated. Significant down-regulation in the proteins of PSI and the levels of phycobiliproteins were observed in *Synechococcus* sp. exposed to high light stress (Xiong et al., 2015b). Proteomic profiling revealed that under inorganic phosphorus stress, the proteins of PSI and II, ETC and photosynthetic pigments were reduced in *Anabaena* sp. strain 90 (Teikari et al., 2015). This could be due to the ROS mediated bleaching and over-reduction of PSs under energetic UV-B radiation, high light and low temperature thus affecting the photosynthetic reaction resulting in overall reduction in generation of reduced NADPH and ATP.

The limited or diminished supply of ATP and NADPH_2_ may also inhibit the CO_2_-fixing ability. CO_2_ fixation by CCM is the most important pathway for the synthesis of these bio-products. CCM include the Calvin-Benson cycle that is closely associated with photorespiratory 2-phosphoglycolate (2PG) metabolism, glycolysis, oxidative pentose phosphate (OxPP) and TCA cycle. Excess organic carbon (i.e., glucose) that is not instantly used for growth and metabolism can be stored as glycogen (storage form of organic carbon). Abiotic stresses severely affect these CCM eventually halt the growth and metabolism of cyanobacteria. Several proteins associated with CCM and thus involved in fulfilling the energy demand of the cells are the primary target of the abiotic stress. We found that the expression of phosphoribulokinase was down-regulated under UV-B stress (Babele et al., 2015). This enzyme catalyzes the conversion of ribulose-5-phosphate into ribulose-1, 5-phosphate during the process of CO_2_ assimilation. Many energy metabolic enzymes, i.e., aminomethyltransferase, carbamoyl-phosphate synthase large subunit, GDP-mannose pyrophosphorylase, phosphoglycerate mutase, and OxPP Cycle gene were down-regulated upon prolonged UV-B exposure to *Synechocystis* sp. (Gao et al., 2009). Glucokinase and glyceraldehydes 3-phosphate dehydrogenase-2 showed reduced levels in methyl viologen treated *Anabaena* PCC7120 cells (Panda et al., 2014). Enzymes of Calvin cycle [ribulose 1,5-bisphosphate carboxylase/oxygenase (RuBisCO) large subunit, and phosphoribulokinase], pentose phosphate pathway (6-phosphogluconate dehydrogenase, transketolase and fructose bisphosphate aldolase) and glycolysis (glucose-6-phosphate isomerase, glyceraldehyde 3-phosphate dehydrogenase, fructose-1,6-bisphosphate aldolase, and phosphoglycerate kinase) were depressed in three *Anabaena* species exposed to cadmium (Singh et al., 2015). It has been demonstrated that RuBisCO might show structural modifications this could be the results of photodegradation, fragmentation and denaturation, active site modifications and solubility of membrane proteins (Feller et al., 2008). Down-regulation in these enzymes suggest noticeable inhibition of photosynthetic dark reaction in *Anabaena* species and their inability to generate ATP, which is used for maintaining metabolic processes under stressful condition. Furthermore, inhibition in the activity of Kreb’s cycle enzymes in all three studies *Anabaena* species not only lowers the generation of ATP and NADPH but also reduce the production of many other metabolic precursors of different metabolic pathways. It was also reported that under the stress of inorganic phosphorus there was a reduction in the protein level of large and small subunit of RuBisCO in *Anabaena* sp. strain 90 (Teikari et al., 2015). Transketolase connects the pentose phosphate pathway to glycolytic intermediates (Schenk et al., 1998; Jung et al., 2004). Its down-regulation might be due to its sensitivity to accumulated ROS in the cells and competitive inhibition of TK catalyzed reactions (Kruger and von Schaewen, 2003; Singh et al., 2015). In the Calvin cycle FBA II reversibly catalyze the conversion of fructose 1, 6-bis phosphate (FBP) into glyceraldehydes 3-phosphate (GAP) and dihydroxy acetone 3-phosphate (DHAP) (Nakahara et al., 2003). During stress, GAP and FBP might be converted into glucose 6-phosphate for re-entry into the PPP for NADPH synthesis and FBA also causes RuBP regeneration (Fridlyand and Scheibe, 1999). Another Calvin cycle enzyme, i.e., fructose 1,6-bisphosphatase (FBPase) increases the production of fructose 6-phosphate and glucose by catalyzing the conversion of fructose-1,6-bisphosphate (Yan and Xu, 2008). Thus down-regulation in the activity of FBPase indicates the decline in carbon fixation, NADPH/NADH production and therefore the loss in cell growth (Gao et al., 2009). The loss in the activity of all these enzymes were reported in different *Anabaena* sp. under temperature (Mishra et al., 2009) and UV radiation stress (Shrivastava et al., 2015). Subcellular proteomic profiling of *Spirulina* sp. under low-temperature stress indicate that the expression levels of phosphoenolpyruvate synthase and phosphoenolpyruvate carboxylase were repressed (Hongsthong et al., 2008). The enzyme phosphoenolpyruvate synthase catalyzes the essential step in Calvin cycle and converts pyruvate into phosphoenolpyruvate (PEP). Gluconeogenesis occurs in the cells when pyruvate or lactate is used as a carbon source. PEP can also be utilized as an energy source for the phosphotransferase system of sugar uptake. Phosphoenolpyruvate carboxylase plays an antipleurotic role in bacteria; it supplies oxaloacetate to the TCA cycle, which requires an uninterrupted supply of C4 molecules, so as to fulfill the demand of intermediate molecules which are consumed in amino acid biosynthesis.

Defects in the proteins/enzymes of carbon metabolism also negatively affect the protein machinery of nitrogen metabolism. Comparative proteomic analyses of three *Anabaena* species under cadmium toxicity indicate defects in nitrogen metabolism. A key enzyme, i.e., glutamine synthetase (GlnA) of nitrogen assimilation which catalyzes the production of glutamine from glutamate and ammonia is severely down-regulated across three *Anabaena* species. Another protein involved in reassembly of intracellular membranes during heterocyst differentiation and nitrogen fixation, i.e., putative heterocyst to vegetative cell connection (FraH) showed a decline in all three *Anabaena* species upon cadmium exposure (Singh et al., 2015). Enzymes of nitrogen metabolism such as nitrogenase, nitrate, and nitrite reductases are severely affected by UV radiations and other abiotic stresses. Nitrogenase has been found to be extremely sensitive to UV-B radiation (Kumar et al., 2003).

### Enzymes of RNA and Protein Biosynthesis and Transport

One of the important classes of down-regulated proteins is involved in the transcription and translation process. Defects in the CCM leads to drastic down-regulation of many proteins/enzymes of transcription and translation due to the limited source of energy. Many transcriptional proteins such as thymidine kinase, oligoribonuclease, exodeoxyribonuclease VII small subunit, and phosphoribosylformylglycinamidine synthase I (FGAM synthase) and translational protein, i.e., ATP-dependent Clp protease proteolytic subunit 2 down-regulated many folds in *S. platensis* upon cold stress (Jeamton et al., 2008). A transcriptional regulator (abrb family) protein is also down-regulated in *S. plantensis*-YZ under salt-stress conditions. Proteomic analysis of *Synechocystis* sp. following prolonged UV-B irradiation showed down-regulation in transcriptional (ribonuclease D) and translational (methionine sulfoxide reductase A) proteins. Proteome analysis of three *Anabaena* species describe that ribosomal proteins and other translational proteins such as EF-Tu, EF-Ts, EF-G, RPS-1, and Pmb-A were significantly down-regulated upon cadmium exposure in all the *Anabaena* species. These proteins are associated in proper ribosome assembly, post-transcriptional processes and translation indicating cadmium mediated obstruction in protein biosynthesis by inducing degradation and alteration in the tertiary structure of proteins (Singh et al., 2015). A translational protein slr1463, an elongation factor EF-G is down-regulated in *Synechocystis* PCC 6803 under high pH stress (Zhang et al., 2009). Label-free shotgun proteomic analysis of marine *Synechococcus* sp. strain WH8102 describes the down-regulation in several translational, ribosomal biogenesis and transcriptional proteins under low-temperature stress (Varkey et al., 2016). Among these proteins, many ribosomal proteins, RNA binding proteins, translation initiation factors, tRNA synthetases and GTP binding proteins are many folds down-regulated. Proteins such as transcription elongation factor NusA, DNA directed RNA polymerase beta subunit and RNA polymerase sigma factor (type 1) were also down-regulated under low temperature stress. Some of the protein with similar names and functions are also found to be down-regulated in *Synechocystis* sp. under nickel, cobalt and cadmium toxicity (Mehta et al., 2014). Under the long-term UV-B stress in *Synechocystis* sp., the enzymes, i.e., three isoforms of Msr-A, aminopeptidase P, and 30S-RP that are associated in translation were down-regulated (Gao et al., 2009). Msr has two important functions, first, it repairs denatured proteins and maintains the proper functioning of important housekeeping proteins (Ezraty et al., 2004). Second, it helps in the reduction of methionine residues rich proteins thus facilitates these proteins to act as ROS quenchers or sinks by a progressive redox cycle on the surface of methionines which is very important under stressful condition (Stadtman et al., 2003).

## Concluding Remarks and Future Perspectives

The present review deals with the effect of different abiotic stresses on the cyanobacterial proteome. Efficient protein extraction protocols and advanced proteomic tools/techniques for identification of novel/hypothetical proteins have been standardized in different strains of cyanobacteria for better knowledge on the molecular mechanism(s) of abiotic stress sensing and intracellular signal transduction in response to abiotic stresses. Interestingly, 2-DE/MALDI-TOF MS and gel free-LC-MS/MS systems have been exploited for the identification of novel proteins expressed under stress conditions. One of the exciting areas will be investigating the roles of hypothetical proteins up-/down-regulated during stress situations. Their functional characterization will illuminate new mechanisms involved in complex mitigation process. Till date, it is not well known that how cyanobacteria perceive changes in stress that lead to the stress-induced expression of various proteins. Another important question is to how the expression of the genes for stress sensors and transducers occurs (Murata and Suzuki, 2006). Research has mainly focussed on photosynthetic ETS chain as the major source of ROS production, but not much is known about the contribution of the respiratory chain in ROS production. Inside a photosynthetic cell, many types of ROS are produced simultaneously, therefore it is complicated to determine the specific targets of ROS. In future, research should be focussed toward the identification and characterization of the physiological/molecular responses they provoke and specific targets of each ROS. Research should also focus on the characterization of truly specific sensory genes and proteins up and/or down-regulated upon a particular type of abiotic stress. In order to identify and characterize possible sensor protein and their regulatory molecules, systematic analysis of gene’s promoter structure and its potential sigma and transcriptional factors proteins and the role of small, non-coding RNAs (microRNAs) could be a good point to initiate.

**FIGURE 3:**
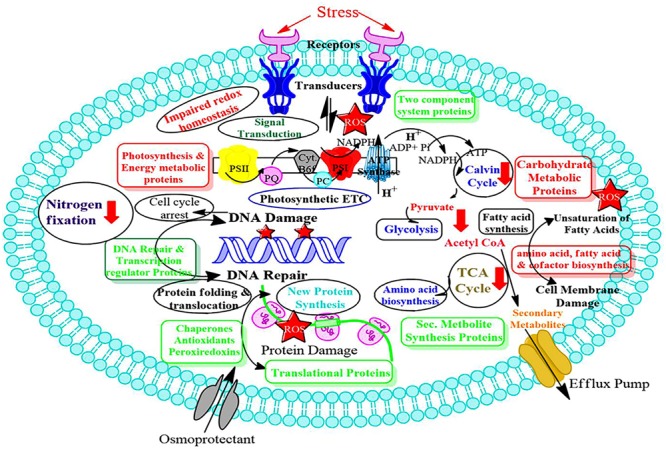
Scheme of abiotic stress responsive protein networks involved in cyanobacteria. Proteins shown in red boxes are down regulated while proteins shown in green boxes are upregulated. Metabolic processes having red arrows are affected by abiotic stresses.

Redox changes taking place inside the photosynthetic organism can affect the effectiveness of biofuel production. Despite the utmost importance of cyanobacteria for future industrial setups, the knowledge of site-specific redox changes are insufficient, especially under light conditions, therefore, critical analysis of whole proteome is urgently required at quantitative and site-specific levels. These understanding will enlighten the specific growth conditions necessary for cyanobacteria to thrive in industrial settings. Cyanobacteria are termed as prominent microbial biofactories for producing biochemicals and biofuels harnessing solar energy and carbon dioxide. Little if any, is known about the specific cysteine sites in the organism, which act as switches, turning redox on and off, especially since light changes from day to night (Guo et al., 2014). With the help of cyanobacterial genomics, and different ‘-omics’ technologies, such as transcriptomics, proteomics, and metabolomics, have been used in cyanobacteria to study acclimation toward different environmental stresses. In this respect, a critical evaluation of the transcriptomic and proteomic data by advanced bioinformatic tools is utmost necessary. These technologies, along with the site-directed mutagenesis will generate the knockouts of the desired genes and allow us to screen genes and their coding proteins as potential sensory polypeptides. These advancements in the field of cyanobacterial research make them feasible to study different types of stress responses. More recently, cyanobacteria are receiving a lot of attention due to their immense application in the production of biofuel and bioremediation. Advanced knowledge of stress mitigation will be beneficial in these areas. It is apparent that in the near future environment will be more polluted and hence harsher than present environment. Therefore, the use of stress tolerant cyanobacteria will be beneficial industrially. We feel that the present review would provide new insights into the cyanobacterial stress responses (Figure 3), which are required for the development of stress-tolerant GEMS for the benefit of humankind. It will also help in studying de-regulation in protein abundance within the cyanobacteria cell in response to abiotic stress.

## Author Contributions

PB wrote the review manuscript. JK and VC corrected the manuscript. All authors approved and revised the final version of the manuscript.

## Conflict of Interest Statement

The authors declare that the research was conducted in the absence of any commercial or financial relationships that could be construed as a potential conflict of interest.
